# Validation and pilot feasibility study of a novel screener to assess diet, lifestyle and mental health in people living with and beyond cancer: Study protocols

**DOI:** 10.1371/journal.pone.0323671

**Published:** 2025-06-05

**Authors:** Alice Chaplin, Janna Wordsworth, Lara Prohens, Antònia Obrador-Hevia, Monica Guillot, Ignacio Ricci-Cabello, Albert Sesé, Dora Romaguera

**Affiliations:** 1 Health Research Institute of the Balearic Islands (IdISBa), Palma, Spain; 2 Consorcio CIBER, M.P. Fisiopatología de la Obesidad y Nutrición (CIBEROBN), Instituto de Salud Carlos III (ISCIII), Madrid, Spain; 3 University Hospital Son Espases (HUSE), Palma, Spain; 4 University of the Balearic Islands, Palma, Spain; 5 CIBER Biomedical Research Center in Epidemiology and Public Health (CIBERESP), Instituto de Salud Carlos III (ISCIII), Madrid, Spain; 6 Department of Psychology, University of the Balearic Islands, Palma, Spain; PLOS: Public Library of Science, UNITED KINGDOM OF GREAT BRITAIN AND NORTHERN IRELAND

## Abstract

**Background and aim:**

Current clinical care may not address behavioural and psychosocial elements which can influence quality of life (QoL) and recurrence risk of people living with and beyond cancer (PLWBC). There is a lack of validated tools to assess diet, lifestyle and mental health in PLWBC. We have developed a screener to identify individuals who may need further support beyond cancer recurrence. The aim is two-fold: 1) validate the screener in PLWBC; and 2) carry out a pilot feasibility study (PFS) to explore the impact of a lifestyle complex intervention (diet, physical activity and mental health components) on the QoL of PLWBC.

**Methods:**

The study will be carried out at the University Hospital Son Espases (Spain) in PLWBC. A face validity study (n = 15) will assess construct interpretation, completion time, and acquiescence of the screener. For construct validity and reproducibility analysis (n = 100), participants will answer the screener together with validated diet, lifestyle, and mental health questionnaires for comparison. Body composition, physical activity, strength and cortisol levels will be assessed using validated instruments. All participants will answer the screener 7–10 days later for reproducibility analysis. Participants will then be randomized (1:1) to the Low Intervention (LI) or the High Intervention (HI) for the PFS study. LI will receive general advice regarding diet, lifestyle and mental health, and HI will receive individual and group sessions with specialised health professionals. Participants will be followed for three months. Primary outcomes include: 1) validity and reproducibility of the screener; and 2) feasibility of a complex intervention to improve QoL of PLWBC. Secondary outcomes include changes in screener answers and body composition.

**Discussion:**

A validated screener which detects PLWBC’s needs could be used in follow-up care plans. The PFS will inform on the recruitment of participants and identify potential shortfalls of the design and efficacy.

**Trial registration:**

ClinicalTrials.gov NCT06582498.

## Introduction

The number of people living with and beyond cancer (PLWBC) is increasing, mainly due to an ageing population and advances in screening programs and treatments [1–3]. Traditionally, follow-up of PLWBC has focused on detection of cancer recurrence or new cancers, but often it does not fully address behavioural and psychosocial elements which could improve quality of life (QoL) and decrease recurrence risk [4,5]. It has been previously suggested that survivorship care guidelines should include screening for common problems and address general survivorship issues [6,7], as well as consider further endpoints other than cancer recurrence, undertaking a more multidisciplinary, holistic healthcare approach [8].

Important work is being developed in this field; for example, the European Organisation for Research and Treatment of Cancer Quality of Life Group has recently published a 73-item questionnaire which evaluates physical, mental and social health-related QoL of cancer survivors [9]. Other similar tools are being developed [10–12]. However, to the best of our knowledge, there is a lack of validated screeners available for both clinical and research purposes which evaluate the main modifiable lifestyle factors, such as body composition, physical activity and diet, among others.

Although evidence is still not strong enough to make specific recommendations for PLWBC, the World Cancer Research Fund and the American Institute for Cancer Research (WCRF/AICR) state that cancer prevention guidelines regarding nutritional and lifestyle factors (body weight, physical activity, diet, alcohol intake and smoking) should be followed after a cancer diagnosis [1]. This is in line with other recommendations published [13]. Studies have shown that cancer survivors have similar rates of obesity, physical inactivity and unhealthy diets than those who have not had cancer [14–17], and that following healthy behaviours can improve survival and QoL [18,19]. Furthermore, PLWBC also often present sarcopenia [20], and report poor sleep quality and varying degrees of psychosocial distress and mental health issues long after finishing their treatment [8,21–24]. Therefore, implementing effective models of care which support patients and takes into consideration these concerns seems to be key in future survivorship care plans [4].

A recent metanalysis evaluated the impact of complex lifestyle interventions, including diet, physical activity and mental health, on the QoL of cancer survivors [25]. It reported that short (<12 weeks) lifestyle interventions improve QoL of these patients; however, few studies addressed mental health particularly and therefore further research is encouraged. We recently developed a screener (under review) which takes into consideration body composition, physical activity and sarcopenia, diet and alcohol intake, smoking, sleeping behaviours and mental health, to detect the needs of PLWBC in clinical settings and improve survivorship care. Briefly, we identified a list of items to include together with an international expert panel using a two-round modified electronic Delphi. Thus, the aim of this study is to determine the validity and reproducibility of the screener. Furthermore, we propose a pilot feasibility study (following previously reported guidelines [26]) to assess the impact of a multimodal intervention (diet, physical activity and mental health) on QoL of PLWBC.

## Materials and methods

### Aims and study design

**Fig 1:**
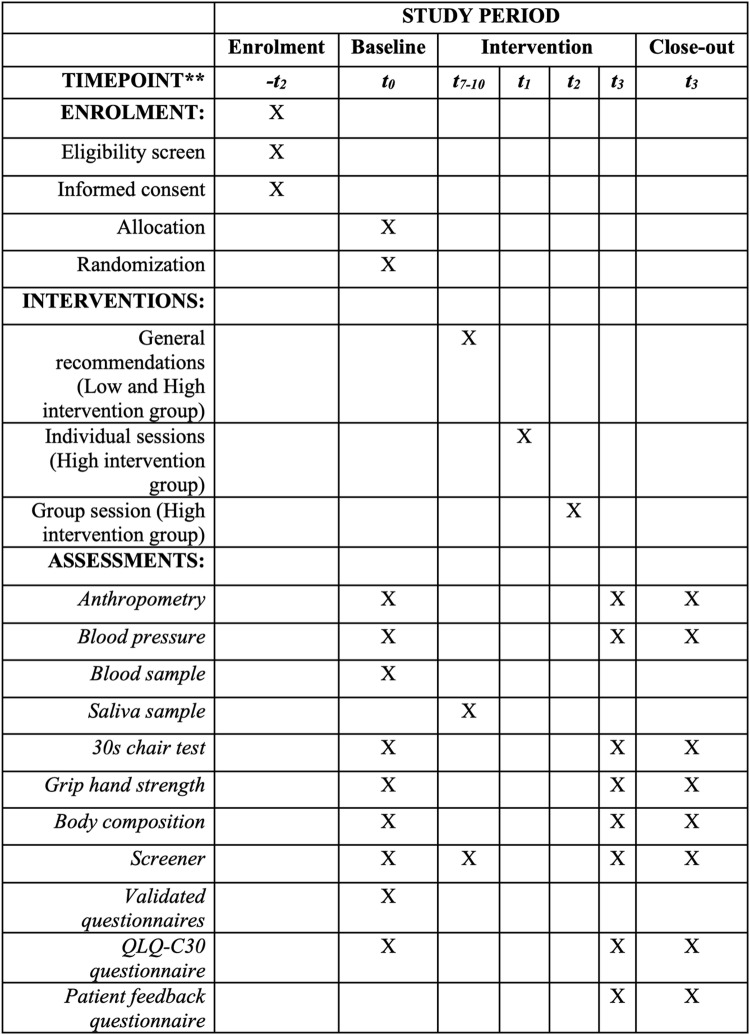
SPIRIT schedule of enrolment, interventions and assessments. **Time-points of the protocol: *-t*_*2*_, two months before baseline; *t*_*0*_, baseline: *t*_*7-10*_, 7–10 days after baseline; *t*_*1*_, 1 months after baseline; *t*_*2,*_ 2 moths after baseline; *t*_*3*_, 3 months after baseline.

This study is divided into two aims:

- Aim 1: Validation of the screener, including a face validity study and a construct validity study. This will enable us to determine its validity, reproducibility, comprehension and acceptability.
- Aim 2: Pilot feasibility study, which will be a small-scale randomized control trial, to determine the feasibility of carrying out a complex lifestyle intervention (diet, physical activity and mental health components) in PLWBC to improve QoL. Participants will be allocated to either the high intervention (HI) or low intervention (LI) arm. A SPIRIT schedule including enrolment, interventions and assessments is included in Fig 1, and a complete SPIRIT checklist can be found in S1 Checklist. This trial has been registered with ClinicalTrials.gov under the number NCT06582498. The total length of this study will be of three months.

The study will be carried out at the Health Research Institute of the Balearic Islands (Palma, Spain) and has been approved by the Ethics Committee of the Balearic Islands (IB5335/23PI; date of approval: 24th April 2024, meeting nº: 04/2024). A copy of the protocol presented to the Ethics Committee can be found in S1 Protocol. Any modifications to the protocol will require a formal amendment, which will be reviewed and approved by the Ethics Committee of the Balearic Islands prior to implementation. The funding source was not involved in any aspect of the study’s design, research objectives, or methods, and will not participate in any phase of the study’s execution (data collection, management, or oversight of study procedures), data analysis, or publishing of results.

**Fig 2:**
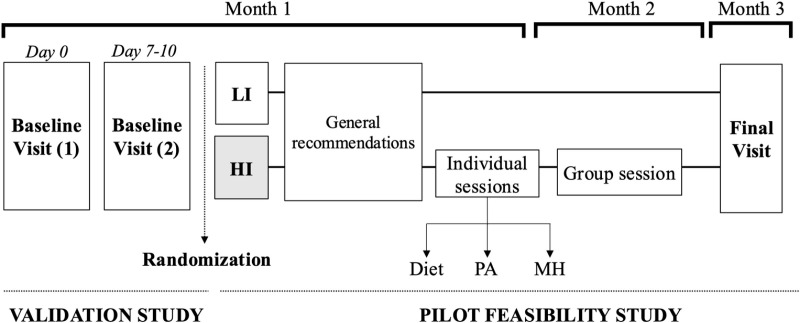
Scheme of the validation study and the pilot feasibility study. HI: High intervention; LI: Low intervention; MH: Mental health; PA: Physical activity.

A scheme of the Validation and Pilot Feasibility Study can be found in Fig 2.

### Inclusion and exclusion criteria

The pre-defined criteria for participation in the study are described in Table 1.

**Table 1:** Eligibility criteria of participants.

Inclusion criteria	Exclusion criteria
≥ 18 years old Men and women Able to read and understand Spanish Prior diagnosis of cancer (stages I to III) Have completed all systemic treatment (surgery, chemotherapy, radiation therapy) at least ≥3 months prior to the study start date, and no later than 5 years Able to attend at least three visits	Ongoing cancer Have a debilitating medical or psychiatric illness Present a disorder that compromises comprehension (e.g., dementia) Pregnancy/breastfeeding

### Recruitment and sample size

The recruitment of patients started on the 23/05/2024 and is expected to end in March 2025. Data collection started on 02/09/2024 and is expected to end in June 2025.

#### Face validity study participants.

Patients (n = 15) attending the Spanish Association Against Cancer (AECC, in Spanish) will be recruited and informed of the face validity study by health workers at the Palma (Spain) branch.

#### Construct validity and pilot feasibility study participants.

PLWBC (n = 100) will be recruited by the Oncology and Hematology Departments at the University Hospital Son Espases (HUSE) located in Palma, Spain. Patients will be briefed by their treating physician and, if they are interested, they will be provided with a study summary and contacted by a member of the research team. During this preliminary interview, either by phone or face-to-face, potential participants will be informed in detail of the study. A record will be kept of non-participation, considering both patients who are ineligible or decline to participate. All patients will be required to read and sign a written informed consent during the Baseline Visit (1), and will be informed that they are free to withdraw from the study at any point without giving any reason and with it having no impact on their usual care. If a participant decides to no longer participate, a signed document will be requested to confirm withdrawal and whether they consent use of the data collected until that point. Gender balance will be accounted for and actively considered during recruitment.

Given the short nature of this pilot study, major changes in the primary outcome (QoL) are not expected and hence type II error is not a major concern. Accepting an alpha risk of 0.05 and a beta risk of 0.2 in a two-sided test, 45 subjects would be necessary in the HI and 45 in the LI to detect statistically significant differences greater than or equal to 6 units (corresponding to 10% change). The common standard deviation is assumed to be 9 (based on a study reporting a mean QLQ-30 of 60, SD 9.3 points over 100 [27]). A drop-out rate of 20% is anticipated. There is a lack of consensus regarding how to compute the sample size in validation studies, with recommendations ranging from 2 to 20 subjects per item and sample sizes ranging from 50 to 250 [28]. Thus, based on the literature, and assuming a 20% lost to follow-up, we will recruit 120 participants. This is a feasible number given the expected cancer survivors visiting the hospital in 1 year.

### Validation study

#### Face validity.

A first version of the screener will be sent to a convenience sample (n = 15) via text or email using an online platform (Jotform), together with a short ad hoc questionnaire (S1 Table) to determine comprehension, time to complete, construct interpretation and acquiescence (apparent validity). Data obtained will allow for refinements.

#### Construct validity and reproducibility.

**Baseline visit (1):** Participants (n = 100) will be recruited at HUSE and invited to the Clinical Trials Unit at IdISBa (Palma, Spain). All participants will be handed an information sheet and an informed consent to sign before starting the study. First, participants will answer the screener (self-administered) using an online platform (Jotform). After answering the screener, data on body composition, physical activity, diet, strength, blood pressure and mental health will be collected using validated methods (see below –4.2.2. and 4.2.3.). To test the reliability and construct validity of the screener, results will be compared to those obtained using validated methods for each construct.

**Validated questionnaires:** Questionnaires will be self-administered, except for the food frequency questionnaire (FFQ), which will be administered by a trained dietitian. All questionnaires are available in Spanish.

- Quality of life: EORTC Quality of Life for cancer patient’s questionnaire (QLQ-C30) [29].
- Sarcopenia: Strength, assistance with walking, rising from a chair, climbing stairs, and falls (SARC-F) questionnaire [30].
- Physical activity: International Physical Activity Questionnaire (IPAQ) [31].
- Fatigue: Fatigue Assessment Scale (FAS) [32].
- Diet: An adapted version of the validated, semi-quantitative, 143-item FFQ for the Spanish population [33–35] will be used, together with the Mediterranean Diet Adherence Screener (MEDAS) [36].
- Smoking: Questions to assess smoking will be based on the TackSHS project [37].
- Sleep: Pittsburgh Sleep Quality Index (PSQI) [38].
- Stress: Perceived Stress Scale (PSS) [39].
- Mental health: Patient Health Questionnaire (PHQ-9) [40].
- Social support: Multidimensional Scale of Perceived Social Support (MSPSS) [41].

**Validated measurements:** The following measurements will be carried out by trained researchers and health professionals at the Clinical Trials Unit during the Baseline Visit (1).

- Anthropometric measurements: Body weight (kg) and composition will be measured in light clothes and without shoes (inBody 770 (Biospace, Seoul, Republic of Korea)). Height (cm) will be measured in duplicate using a wall-mounted stadiometer. Body mass index (BMI) will be calculated as weight (kg)/height (m)^2^. All participants will receive a copy of their body composition analysis at the beginning, and will receive one at the end of the study. Waist circumference (cm) will be determined in duplicate midway between the lowest rib and the iliac crest using a measuring tape. Hip circumference (cm) will be determined in duplicate at the most prominent point of the trochanter bone with a measuring tape.
- Blood pressure (BP): BP will be measured in triplicate after asking the participant to rest for 5 min (sitting down) using a semi-automatic oscillometer (Carescape^TM^ V100, GE Health Care).
- Strength: Grip strength will be measured using a hand-held dynamometer (model TKK-5001, Logro Hispania, Spain). Participants will be asked to stand up in a relaxed position with both arms extended to the side of the body. Strength will be measured using both arms alternately in triplicate.
- Lower-limb muscle strength will be determined using the 30-s Chair Stand test [42], which will be carried out by trained dietitians as described in the previously published protocol.
- Physical activity, sedentary behaviour and time in bed: Participants will be asked to wear an accelerometer (GENEActiv, ActivInsights Ltd., Kimbolton, UK) on their non-dominant wrist continuously for 7–10 days.
- Stress: Cortisol levels will be determined in saliva. Briefly, participants will be given three storage tubes and three oral swabs from Salimetrics (bio Nova científica, Madrid, Spain), together with instructions, to collect saliva samples at home throughout the course of one day: 1) within the first 30 min of waking up; 2) maximum peak of stress of the day; and 3) at the end of the day, when the participant is feeling relaxed. After collecting them, participants will be asked to store them at 4ºC. The following day they will be required to bring them to the Clinical Trials Unit for processing. Samples will be centrifuged and stored at −80ºC until further processing. Samples will be assayed in duplicate for cortisol using a human enzyme-linked immunosorbent assay kit following the protocol specified by Salimetrics (bio Nova científica, Madrid, Spain).
- Other biological samples: Participants will be offered the option to give a blood sample for future analysis. Trained nurses will collect and centrifuge the blood samples for plasma, buffy coat fraction and serum using standard procedures. Samples will be stored at −80ºC until further analysis.

**Other co-variates:** At the Baseline Visit (1), a comprehensive sociodemographic questionnaire will be administered to all participants to collect information on the following: personal data (sex, gender, age, country of origin, race); education and type of work; family (marital status, children, living arrangements); diet (access to food, use of supplements); and clinical history (any known conditions, current and past medications/treatment, menstrual status).

**Baseline visit (2):** All participants will be invited to answer the screener again 7–10 days after the first visit to test the reproducibility of answers (re-test method).

### Pilot feasibility study

Two types of interventions have been designed for this randomised pilot feasibility study. The LI is based upon standard advice offered to PLWBC, whereas the HI will offer participants individual and group visits with a dietitian, a physical activity expert and a psychologist (Fig 2).

#### Randomization.

Allocation to either arm will be done using the Study Randomizer® tool and considers a fixed block size of four. Variables imputed will include sex (male/female), age (<50 years or ≥50 years) and type of cancer (breast or non-breast cancer). The allocation sequence for each participant will be securely stored in a password-protected spreadsheet, accessible only to the research team. Participants will be blinded to the arm they were allocated to. The research team will not blinded due to the personalised nature of the intervention.

#### Low intervention.

During the Baseline Visit (2), participants assigned to the LI arm will receive the standard recommendations available for PLWBC [1,6] and briefly explained by a trained dietitian.

#### High intervention.

During the Baseline Visit (2), participants assigned to the HI arm will receive the same standard recommendations offered to the LI together with a personalized care plan. This will consist of three 45-min appointments with a dietitian, a physical activity expert with expertise in cancer patients and a trained oncology psychologist. These will be delivered at the beginning of the study (Month 1, see **Fig 2**). During Month 2, participants from the HI arm will be invited to a three-hour group session with all three health professionals to discuss further diet, physical activity and mental health.

#### Final visit.

All participants will be invited to a Final Visit (Month 3) to assess the impact of the intervention. Both the screener and the QLQ-C30 [29] questionnaire will be administered to determine changes in lifestyle and QoL. Anthropometric measurements, blood pressure and strength will be measured as described above (4.2.3. *Validated measurements*). Information regarding acceptability and perception of the intervention by participants will be collected using an adapted version of the Research Participant Perception Survey – Long (RPPS-L) Broadcast Spanish version [43].

Throughout the study, adherence will be monitored by tracking which participants attend all appointments and group sessions. If participants miss any sessions, we will record the specific elements of the intervention they did not attend. If a participant decides to completely withdraw from the trial, their decision and corresponding details will be documented.

### Measured outcomes

The primary outcome of the Validation Study will be to determine the validity and reproducibility of the newly developed screener. Apparent and construct validity will be assessed using the validated data collected during the Baseline Visit (1), and reproducibility of the screener will be determined using the re-test method by comparing screener data from the Baseline Visit (1) with that obtained at the Baseline Visit (2).

On the other hand, the primary outcome of the Pilot Intervention Study will be to determine the feasibility of lifestyle intervention to improve QoL in a time frame of three months. The following will be assessed: recruitment rate, attrition, acceptability of the intervention (RPPS-L), adherence and viability. This information will be used to determine a sample calculation for a larger trial and determine limitations and down-falls of the study design. Furthermore, efficacy of the intervention will be determined by comparing QLQ-C30 scores obtained at the Baseline and Final Visits.

The secondary outcomes include changes in Life S-Can answers and body composition.

### Data management plan

A data monitoring committee will not be established; however, a comprehensive data management plan is in place to ensure the integrity and security of the trial data.

No datasets have been generated or analysed at present. All data obtained will be integrated into an electronic, password-protected database. A data management plan following the FAIR principles has been established. The treatment, communication and transfer of personal data of all participating subjects will be in accordance with the provisions of Organic Law 3/2018, of December 5, on the protection of personal data and guarantee of digital rights. Participants’ data and biological samples will be identified by means of a code and only the study researchers will have access to them. All data collected by means of forms and questionnaires will be pseudoanonymous, whereby the participant will only have to indicate their individual code and will not be asked for identifying variables. All records with names or other personal identifying information will be kept separately from study records with ID numbers. The datasets generated and/or analyzed in this study will not be publicly available, but can be available on request. In the case of biological samples (saliva, blood), these will be obtained and stored according to what is established in Law 14/2007 3rd July of Biomedical research. Biological samples will be stored in a sample collection (ref. C0008029) for 10 years.

### Analysis

Normality of data will be assessed using standard tests, such as Shapiro-Wilk. For validation and reproducibility analysis, agreement between data from the screener and validated methods will be determined using a range of statistical tests: cross-classification; Cohen’s weighted kappa statistic; Spearman correlation coefficient; linear regression model; intraclass correlation coefficient (ICC); and/or the Bland-Altman method. Analyses will be carried out using Stata (StataCorp LLC).

To determine the feasibility of the pilot intervention in changing QoL, between-group (HI vs. LI) analysis of continuous and discreet variables will be carried out with data from the Final Visit. Before-and-after analysis of each group will also be carried out by comparing data from Baseline Visit (1) vs. Final Visit. Statistical tests include independent t-tests and chi-squared tests, or the equivalent non-parametric tests, according to normality of the data.

### Patient and public involvement

Patients will be involved in both the Validation Study and the Pilot Feasibility Study. On one hand, patients from the AECC (Palma, Spain) will be invited to participate in the Face Validity Study, which aims to obtain qualitative data on questionnaire comprehension, interpretation of the screener, acquiescence (tendency to choose a response regardless of the context) and time to complete the questionnaire. Their involvement at this stage will allow us to refine the screener in the initial stages. Furthermore, by administering the RPPS-L at the end of the Pilot Feasibility Study, we will obtain information on the experience, motivation for participation and satisfaction with the research experience.

### Dissemination of results

Results derived from the validation of the screener will be published in peer-reviewed scientific journals and presented at relevant scientific conferences. The trial results will also be reported in publicly accessible clinical trial databases (https://clinicaltrials.gov/). Furthermore, once they have been published, results will be shared with participating physicians and patients who specifically asked to be informed of the results. This will be done through summary reports or direct updates.

## Discussion

Cancer can have a lasting impact on patients, affecting daily activities and QoL long after treatment has ended [44]. Care of PLWBC is evolving, and it is becoming clear that it should be multifactorial and not be centred only on recurrence risk and survival. However, due to time constraints and clinical burden, health professionals often cannot assess lifestyle and QoL adequately. The present studies aim to validate a screener which could effectively evaluate these components in a timely manner, and test the feasibility of a three-month complex intervention in PLWBC.

### Aim 1: Validation study

There is a range of validated instruments to measure the impact of cancer on QoL, such as the Impact of Cancer (IOC) scale [45], the EORTC Quality of Life cancer survivorship core questionnaire [9] and the QLQ-C30 [29], and the Functional Assessment of Cancer Therapy (FACT-G) scale [46], among others. Other components, such as cancer-related fatigue [11], are also being considered when developing tools for PLWBC. However, to the best of our knowledge, there is no screener available which considers the main lifestyle components in one same questionnaire, including body composition, physical activity, diet, smoking, sleeping habits and mental health, which could be affected in PLWBC. Taking this into account, we have recently developed a rapid screener (under review) to assess individual patient’s needs with the aim for it to be a first step in designing personalised and effective follow-up care plans, and thus promote discussions between patients and clinicians on issues which may not have otherwise been addressed. Its validation is essential to determine its comprehension and acceptability, its capacity to measure what is intended, and its reproducibility. This will ultimately mean that the screener will correctly identify those patients who need further support and standardise its use throughout clinical and research settings.

### Aim 2: Pilot feasibility study

Although patient-centred interventions including multiple components are effective in changing lifestyle behaviours [25], there is still an increasing need for targeted health education interventions for cancer survivors which include modifiable lifestyle factors (such as diet, physical activity) and that also consider other less well-studied domains (sleep, mental health). The aim of the Pilot Feasibility Study is to contribute to the growing body of knowledge regarding the impact of a lifestyle intervention in PLWBC and obtain feasibility indicators [47] for its implementation in our local clinical setting. The study will be carried out over 12 weeks, which is in line with previous studies [25], and collect information on recruitment of participants and attrition rate, acceptability of the study, adherence and viability. Furthermore, it will also give us insight into the impact of a low (general recommendations) vs. high intervention (personalised sessions) and whether the newly developed tool is able to discriminate changes in the abovementioned components. All of this will allow us to define a future larger-scale implementation-effectiveness study, with a higher number of participants and a longer follow-up period to determine whether changes in lifestyle and QoL are sustained, and help identify potential shortfalls of the design and effectiveness in improving QoL.

### Strengths

Patients will be involved where possible in the design and assessment of our studies. Specifically, PLWBC will be consulted on screener items during the Validation phase, and at the end of the Pilot Feasibility Study will provide information on their experience and how to improve a future study design. Furthermore, for both studies, an n = 100 participants is considered, which is in agreement with what is recommended for validation studies [28] and pilot studies [48]. Finally, a wide range of data will be collected, including objective measurements for body composition, strength, physical activity, stress levels and sleeping behaviour; data on nutrition, fatigue, physical activity levels, pain, smoking habits and mental health data by means of validated questionnaires; and blood samples which offer a range of possibilities (biomarkers, liquid biopsy, pharmacogenetics).

### Limitations

The Pilot Feasibility Study will include up to six visits to the Clinical Trials Unit in the time-space of three months, which could become burdensome to some participants. Thus, to ensure patient commitment to the study and reduce travelling to the Clinical Trials Unit, visits will be coordinated (when possible) with other clinical appointments with their care team at the hospital. On the other hand, results obtained from complex interventions make it difficult at the end of the study to identify which intervention components were key in providing significant effects.

### Future steps

Regarding the screener developed, a scoring system (such as a traffic light code or similar) will be devised to aid personalised interventions in a clinical setting, whereby specific recommendations can be given according to the score obtained. Furthermore, a qualitative study will be carried out to explore the perceptions of patients and health professionals on the screener and identify the barriers and facilitators of its implementation in a range of settings (hospital, primary care and/or patient organizations, for example). Briefly, the qualitative study will include focus groups and semi-structured interviews. Factors such as delivery mode, setting, stakeholders involved, expected outcomes and significance of the tool will be assessed. The involvement of patients in the qualitative study, together with the information collected at the end of the Pilot Feasibility Study, will help guide a future intervention study. This study will be based on the ORBIT model [49] for developing behavioral treatments for chronic diseases as well as on the Medical Research Council guidance [50] for evaluating complex interventions.

## Conclusion

A validated screener which considers lifestyle factors, such as body composition, fatigue and physical activity, diet and alcohol use, smoking, sleeping habits and mental health, could aid in identifying PLWBC’s needs and could be used in follow-up care plans to work towards improvement in QoL. The Pilot Feasibility Study will inform on the feasibility of the methods and study design proposed and identify potential shortfalls.

## Supporting information

S1 Checklist Complete SPIRIT checklist. (DOC)

S1 Protocol Project protocol submitted and approved by the Ethics Committee of the Balearic Islands. (DOCX)

S1 Table Ad hoc questionnaire used for the face validity study. (DOCX)
