# Supplementary material for: Validation and pilot feasibility study of a novel screener to assess diet, lifestyle and mental health in people living with and beyond cancer: Study protocols
Source: PLoS One. 2025 Jun 5;20(6):e0323671. doi: 10.1371/journal.pone.0323671 (PMC12140243; doi:10.1371/journal.pone.0323671)
Supplement: S1 Protocol — (DOCX) [file pone.0323671.s002.docx]

**Supporting Information S2. Project protocol submitted and approved by the Ethics Committee of the Balearic Islands.**

**Design of a tool (Life S-Can) to evaluate individual needs after cancer and improve quality of life of cancer survivors**

***Version 2 (22.03.2024)***

**Promotor: IdISBa**

## Principal investigator: Dra. Alice Chaplin Scott

**Collaborating researchers:**

- Dr. Dora Romaguera Bosch (NUTRECOR PI, IdISBa)
- Dr. Albert Sesé
- Dr. Ignacio Ricci
- Dr. Mónica Guillot
- Dr. Antònia Obrador

**Index**

1. Abstract………………………..……………………………………… 3

2. Background and state of the art ……………..…………………………4

3. References …………………………………………………………….. 4

4. Hypothesis ……………..……………………………………………… 6

5. Aims ……..……………………………………………………………..6

6. Type of study …………………………………………………………. 7

7. Materials and methods ………………………………………………....7

8. Legal and ethical aspects ……………………………………………. 11

1. **Abstract**

Project code: N/A

Promotor: Fundació Institut d’Investigació Sanitaria Illes Balears (IdISBa)

Principal investigator: Dr. Alice Chaplin Scott

Centre where the study will be carried out: IdISBa/University Hospital Son Espases

The cancer survival rate is increasing, raising the need to create high-quality survivorship care plans. Often, follow-up of cancer survivors does not fully address behavioral and psychosocial elements that could improve the quality of life of these patients and decrease the risk of recurrence. Currently, there is a lack of brief, validated tools to assess diet, lifestyle, and other behaviors that could influence quality of life and prognosis in cancer survivors. The hypothesis of this project is that the creation of a screener with the capacity to quickly, validly and effectively evaluate nutritional factors, lifestyle, sleep quality and the psychosocial dimension in cancer survivors could be useful in identifying people who may need further care and support. The aim is to create a valid and rapid tool (Life S-Can) to assess a total of seven dimensions (body composition, physical activity, diet, alcohol intake, smoking, sleep behavior and psychosocial factors) in cancer survivors for use in a clinical setting to improve survivorship care and quality of life. The project is divided into four phases and will be carried out at the Son Espases University Hospital (HUSE) (Palma). The objective of Phase I is to design the Life S-Can questionnaire in English together with an international and multidisciplinary panel of experts in cancer, nutrition, epidemiology, primary care, psychology and psychometrics. This first version will be administered to a small convenience sample (n=20) to determine understandability, clarity, and usefulness. In Phase II, Life S-Can will be validated in a total of 100 cancer survivors at HUSE. To do this, patients will be recruited by the Oncology Department and will be invited to go to the Clinical Trials Unit for a comprehensive evaluation. A dietitian will administer Life S-Can and then collect data using validated assessment methods (using validated questionnaires and objective measurements) to compare with data obtained with the Life S-Can. Phase III consists of a pilot intervention study, where participants recruited in Phase II will be randomized to the intervention group or control group. Participants in the intervention group will be offered visits with a health professional (dietitian, physical activity and/or psychology), based on their responses to the Life S-Can, who will provide advice over 3 months. Phase IV is a qualitative study conducted in cancer patients and healthcare professionals (n=40) to obtain data to future design a complex intervention to test the feasibility and effectiveness of implementing Life S-Can in a clinical setting. The care of cancer survivors must be multifactorial; however, often, due to time constraints and clinical burden, healthcare professionals may not adequately assess certain aspects relevant to the quality of life of cancer survivors, such as nutritional status, diet and physical and mental health. Life S-Can will enable doctors, healthcare professionals, charities and other stakeholders to work with cancer survivors from the outset and refer them to appropriate mental health and care teams and resources.

1. **Background and state of the art**

The number of cancer survivors is increasing substantially, mainly due to an ageing population and advances in screening programs and treatments^1,2,3^. Traditionally, follow-up of cancer survivors has focused on detection of cancer recurrence or new cancers, but often it does not fully address behavioral and psychosocial elements which could improve quality of life (QoL) and decrease recurrence risk^4^, and thus may miss important survivor concerns and needs^5^. Therefore, implementing more effective models of care which support patients and detect their needs or after-effects is key in future survivorship care plans^4^. In terms of nutritional and lifestyle factors (body weight, physical activity, diet and alcohol intake), the WCRF/AICR state that, although evidence is still not strong enough to make specific recommendations, cancer survivors should follow cancer prevention guidelines^1^. Furthermore, studies show that cancer survivors often report poor sleep quality and can suffer from varying degrees of psychosocial distress and mental health issues^6,7,8,9,10^.

The Principal Investigator (PI), together with her supervisor, recently developed a short screener to assess adherence to the 2018 WCRF/AICR recommendations for cancer prevention (ongoing); however, it only contemplates body weight, physical activity, diet, alcohol and breastfeeding, and does not consider other dimensions relevant for cancer survivor’s QoL, such as other consideration of body composition beyond obesity (for example sarcopenia, which is highly prevalent in cancer patients and survivors^11^), sleep quality or psychosocial distress. It has been previously suggested that survivorship care guidelines should include screening for common problems and address general survivorship issues^12^, as well as consider further endpoints other than cancer recurrence, such as improvement of health-related behaviors and overall QoL^9^. Efforts have been made towards this objective^13^; however, there is still a lack of short, validated screeners to assess diet, lifestyle, physical health (sarcopenia) and mental and psychosocial concerns as a whole for cancer survivors, which reflect current recommendations for this population and undertake a more multidisciplinary, holistic health-care approach. Thus, a validated and rapid tool which could effectively operationalize all these factors and determine adherence to current guidelines with the ability to identify individual needs, could improve survivorship care and individual risk prediction. Similar tools have been shown to be useful for other conditions, such as Life’s Essential 8^14^, by assisting individual patients and health care teams in cardiovascular health assessment and progress tracking.

Thus, the overall goal of this proposal is to develop and validate a screener (Life S-Can) based on the current dietary and lifestyle recommendations for cancer survivors, as well as to carry out a qualitative study to help design a complex intervention in the future to test the feasibility and effectiveness of implementation of Life S-Can in clinical settings. This tool would enable clinicians work together with cancer survivors early-on, which often need continued support after surviving a cancer, and screen those patients who may need referral to other health and mental care teams and resources. Such a tool could improve survivorship care, QoL and may decrease recurrence risk.

1. **References**
2. World Cancer Research Fund/American Institute for Cancer Research. Diet, Nutrition, Physical Activity and Cancer: a Global Perspective. Continuous Update Project Expert Report 2018. Available at dietandcancerreport.org
3. Miller KD, Nogueira L, Devasia T, Mariotto AB, Yabroff R, Jemal A, et al. Cancer treatment and survivorship statistics. CA Cancer J Clin 2022; 72: 409-36.
4. Parry C, Kent EE, Mariotto AB, Alfano CM & Rowland JH. Cancer survivors: a booming population. Cancer Epidemiol Biomarkers Prev 2011; 20: 1996-2005.
5. Jefford M, Howell D, Qiuping L, Lisy K, Maher J, Alfano CM et al. Improved models of care for cancer survivors. Lancet 2022;399 (10334): 1551-60.
6. de Rooij BH, Park ER, Perez GK, Rabin J, Quain KM, Dizon DS et al. Cluster analysis demonstrates the need to individualize care for cancer survivors. Oncologist 2018; 23(12): 1474-81.
7. Dai S, Mo Y, Wang Y, Xiang B, Liao Q, Zhou M et al. Chronic stress promotes cancer development. Front Oncol 2020; 10: 1492.
8. Divani A, Heidari ME, Ghavampour N, Parouhan A, Ahmadi S, Charan ON et al. Effect of cancer treatment on sleep quality in cancer patients: a systematic review and meta-analysis of Pittsburgh Sleep Quality Index. Support Care Cancer 2022; 30: 4687-97.
9. Alanazi MT, Alanazi NT, Alfadeel MA, Bugis BA. Sleep deprivation and quality of life among uterine cancer survivors: systematic review. Support Care Cancer 2022; 30(3): 2891-2900.
10. Mitchell AJ, Ferguson DW, Gill J, Paul J, Symonds P. Depression and anxiety in long-term cancer survivors compared with spouses and healthy controls: a systematic review and meta-analysis. Lancet Oncol. 2013 Jul;14(8):721-32
11. Mehnert-Theuerkauf A, Hufeld JM, Esser P, Goerling U, Hermann M, Zimmermann T, Reuter H, Ernst J. Prevalence of mental disorders, psychosocial distress, and perceived need for psychosocial support in cancer patients and their relatives stratified by biopsychosocial factors: rationale, study design, and methods of a prospective multi-center observational cohort study (LUPE study). Front Psychol. 2023 Apr 20;14:1125545.
12. Cao A, Ferrucci LM, Caan BJ, Irwin ML. Effect of Exercise on Sarcopenia among Cancer Survivors: A Systematic Review. Cancers (Basel). 2022 Feb 3;14(3):786.
13. National Comprehensive Cancer Network [Internet]. Survivorship Care for Healthy Living [cited 2023 Jul 11]. Patient resources. Available from: [https://www.nccn.org/patientresources/patient-resources](https://www.nccn.org/patientresources/patient-resources).
14. McDonough AL, Lei Y, Kwak AH, Haggett DE, Jimenez RB, Johnston KT et al. Implementation of a Brief Screening Tool to Identify Needs of Breast Cancer Survivors. Clin Breast Cancer. 2021 Feb;21(1):e88-e95.
15. American Heart Association. Life's Essential 8. Healthy Lifestyle.2023. Accessed May16, 2023.https://www.heart.org/en/healthy‐living/healthy‐lifestyle/lifes‐essential‐8.
16. Anthoine E, Moret L, Regnault A, Sébille V, Hardouin JB. Sample size used to validate a scale: a review of publications on newly-developed patient reported outcomes measures. Health Qual Life Outcomes. 2014 Dec 9;12:176.
17. Hulett JM, Fessele KL, Clayton MF, Eaton LH. Rigor and Reproducibility: A Systematic Review of Salivary Cortisol Sampling and Reporting Parameters Used in Cancer Survivorship Research. Biol Res Nurs. 2019 May;21(3):318-334.
18. Wan C, Boileau K, D’Amico D, Huang V, Fiocco AJ, Clément R et al. A cross-cultural analysis of salivary cortisol patterns in breast cancer survivors. Breast Cancer Management 201; 8 (1).[doi.org/10.2217/bmt-2019-0004.](https://doi.org/10.2217/bmt-2019-0004)
19. Campo RA, Light KC, O'Connor K, Nakamura Y, Lipschitz D, LaStayo PC et al. Blood pressure, salivary cortisol, and inflammatory cytokine outcomes in senior female cancer survivors enrolled in a tai chi chih randomized controlled trial. J Cancer Surviv. 2015 Mar;9(1):115-25.
20. **Hypothesis**

The hypothesis is that Life S-Can will enable clinicians, health practitioners, charities and other stakeholders work with cancer survivors early-on to improve survivorship care, QoL and decrease recurrence risk by 1) evaluating adherence to current dietary and lifestyle cancer prevention recommendations, and 2) exploring other concerns relevant for quality of life in cancer survivors, including sleep patterns and psychosocial distress. Life S-Can could be a useful tool in standard patient care, with the capacity to detect behaviors and challenges of survivors and identify the need for further care and support.

1. **Aims**

The increasing number of cancer survivors worldwide, and the evident continued health and mental care support they often require once they have finished treatment, highlights the current lack of short, validated screening tools to determine diet, lifestyle, sleep behaviour and psychosocial distress in this population. The group with whom the PI of the project pertains to has previously developed a short screener to evaluate adherence to the 2018 WCRF/AICR cancer prevention recommendations, which has given the PI in-depth experience in the development and validation of such tools.

**The aim of this project is to develop a valid and rapid screening tool (Life S-Can) with the capacity to evaluate a total of seven domains (body composition, physical activity, diet, alcohol intake, smoking, sleeping behaviour and psychosocial distress) in cancer survivors for its use in a clinical setting to improve survivorship care and QoL.** The project is divided into four specific aims (SA) and will be carried out in one year at the University Hospital Son Espases (HUSE) (Palma, Spain).

**SA1: Develop a short screener (Life S-Can) to evaluate body composition, physical activity, diet, alcohol intake, smoking habits, sleeping behaviour and psychosocial distress in cancer survivors**.

- A1.1. Organize an online international, multidisciplinary expert panel in oncology, nutrition, primary care, psychology, psychometry and epidemiology.
- A1.2. Design the screener in English together with the expert panel: number of questions per domain, instructions needed, examples given and scoring system will be agreed upon.
- A1.3. Translate the screener to Spanish (translation/back-translation method) and culturally adapt it to Spain with the collaboration of the listed Key Personnel.
- A1.4. Upload the screener onto specific software to create a user-friendly tool using external IT services.
- A1.5. Determine comprehension, clarity and usefulness of Life S-Can in cancer survivors using an adhoc questionnaire for the potential refinement of the tool.

**SA2. Determine relative and construct validity of individual domains and total score of the Life S-Can in cancer survivors using data obtained from validated and objective measurements.**

- A2.1. Recruit 100 cancer survivors from the Oncology Department at the HUSE.
- A2.2. Administer Life S-Can to all participants at the Clinical Trials Unit by a trained research dietitian. Re-administer after 7-10 days for reproducibility analysis (test-retest method).
- A2.3. Collect data of individual domains using validated questionnaires and objective measurements.
- A2.4. Data integration and statistical analysis to determine relative and construct validity of Life S-Can.

**SA3. Carry out a pilot intervention study in cancer survivors to determine the feasibility, acceptability and efficacy of Life S-Can.**

- A3.1. Recruit an expert panel in the field oncology specialised in diet, physical activity, lifestyle and cancer psychology, as well as cancer survivors (non-scientific actors).
- A3.2. Design a 3-month intervention plan with the expert panel and non-scientific actors, based on tailored sessions and personalized feedback according to Life S-Can answers, with the aim to improve QoL.
- A3.3. Randomize participants from A2.1. to either the intervention group (IG) or control group (CG). Offer a personalised intervention care plan to patients from the IG, based on their answers and score. Continue with standard care in patients assigned to CG.
- A3.4. Arrange appointments for IG patients at the Clinical Trial Unit with trained oncological dietitians, physical activity specialists and psychologists, depending on their answers (1 to 3 sessions, individual and as a group).
- A3.5. Re-administer Life S-Can and collect data on feasibility, acceptability and efficacy to all participants after 3 months since the first administration of Life S-Can (A2.2).
- A3.6. Data integration and analysis.

**SA4. Carry out a qualitative study in cancer survivors and health professionals to design a complex intervention to determine the feasibility and effectiveness of the implementation of Life S-Can in clinical settings.**

- A4.1. Design a qualitative study in a clinical setting (primary care, hospital) together with Key Personnel listed in the project.
- A4.2. Recruit 40 cancer survivors and health care professionals from the Oncology Department at the HUSE and in Primary Care clinics. Organize four focus groups and semi-structured like in-depth interviews (audio-recorded) of a duration of 1 hour.
- A4.3. Transcribe audios, collate data and analyze using thematic analysis.

1. **Type of study**

The project herein presented proposes four phases: tool development (Phase I); validation of the tool (Phase II - validation study); pilot intervention study (Phase III); and a qualitative study (Phase IV) to design a complex intervention in the future. All three phases and the type of studies carried out in each are described below. For this study to be successful, it will count of the participation of the following key personnel:

- Dr. Alice Chaplin, PI of the project and postdoctoral researcher of the NUTRECOR research group.
- Dra. Dora Romaguera, PI of NUTRECOR research group (IdISBa)
- NUTRECOR team (research dietitians)
- Dr. Albert Sesé
- Dr. Ignacio Ricci
- Dr. Mónica Guillot
- Dr. Antònia Obrador

1. **Materials and methods**

**PHASE 1. TOOL DEVELOPMENT:** The objective of phase I is to design the screener in an estimated period of three months (A1.1. to A.1.5).

**1. Organize an international, multidisciplinary panel of experts:** Dr. Chaplin will gather experts in cancer, nutrition, epidemiology, primary care, psychology and psychometry will be consulted. Local cancer patients’ organisations and charities will also be contacted throughout the project (based on previous collaborations).

**2. Design of screener:** The tool will include no more than 25 questions which cover a total of seven dimensions: body composition, physical activity, diet, alcohol consumption, smoking habits, sleeping behaviour and psychosocial distress (social support, stress, mental health) (Fig.2). The following considerations will be accounted for:

- Originally designed in English and translated to Spanish using the **translation/back-translation method**. Any subsequent changes in the screener will go through the same process (Dr. Chaplin & Dr. Romaguera).
- Questions will be designed in the most standardized manner as possible; however, if examples or additional instructions are necessary, they will be **culturally adapted** to the Spanish population (in a manner that they can be easily adapted to other cultures in the future).
- Possibility of being self-administered and/or administered by a health professional or researcher.
- Easy completion of screener and scoring system: Can be completed without specialised knowledge and scoring system easy to interpret to give **clinical decision support**.
- Upload the screener on an **adequate software** to complete using a phone, tablet or PC.

**3. Administer first version of Life S-Can to a small convenience sample (Study 1):** An n=10 of cancer survivors and n=10 health professionals/researchers (details on recruitment, inclusion and exclusion criteria described below) will be recruited by the Oncology Department of HUSE to complete the first version of Life S-Can, which will be administered by a research dietitian from the research group NUTRECOR. A short adhoc questionnaire will be prepared to administer to both participants and health professional/researchers involved to determine comprehension, clarity, usefulness of the tool, construct interpretation and acquiescence (apparent validity).

**PHASE II. VALIDATION STUDY:** It is expected that this phase will take an estimate of seven months to complete (based on the flow of cancer survivors at the hospital) (A2.1. to A2.4.).

**Study population:** Cancer survivors (n=100) will be recruited by the Oncology Department at the HUSE during routine follow-up visits to the hospital. The study aims to recruit survivors from most prevalent cancer types (i.e. breast, colorectal, lung); however, all cancer types will be considered. There is a lack of consensus regarding how to compute the sample size in validation studies, with recommendations ranging from 2 to 20 subjects per items and sample sizes ranging from 50 to 250 (*Anthoine E, Moret L, Regnault A, Sébille V, Hardouin JB. Sample size used to validate a scale: a review of publications on newly-developed patient reported outcomes measures. Health Qual Life Outcomes. 2014 Dec 9;12:176*). Thus, based on the literature, and assuming a 20% lost to follow-up, we will recruit 120 participants. This is a feasible number given the expected cancer survivors visiting the hospital in 1 year. **Inclusion criteria:** Adults ≥ 18 years old; men & women; able to read and understand Spanish; prior diagnosis of cancer (stages I to III); have completed all systemic treatment (surgery, chemotherapy, radiation therapy) at least ≥6 months prior to the study start date; be currently considered cancer free. **Exclusion criteria:** Ongoing cancer or cancer treatment; have a debilitating medical or psychiatric illness; presenting a disorder that compromises comprehension (e.g., dementia); pregnancy/breastfeeding.


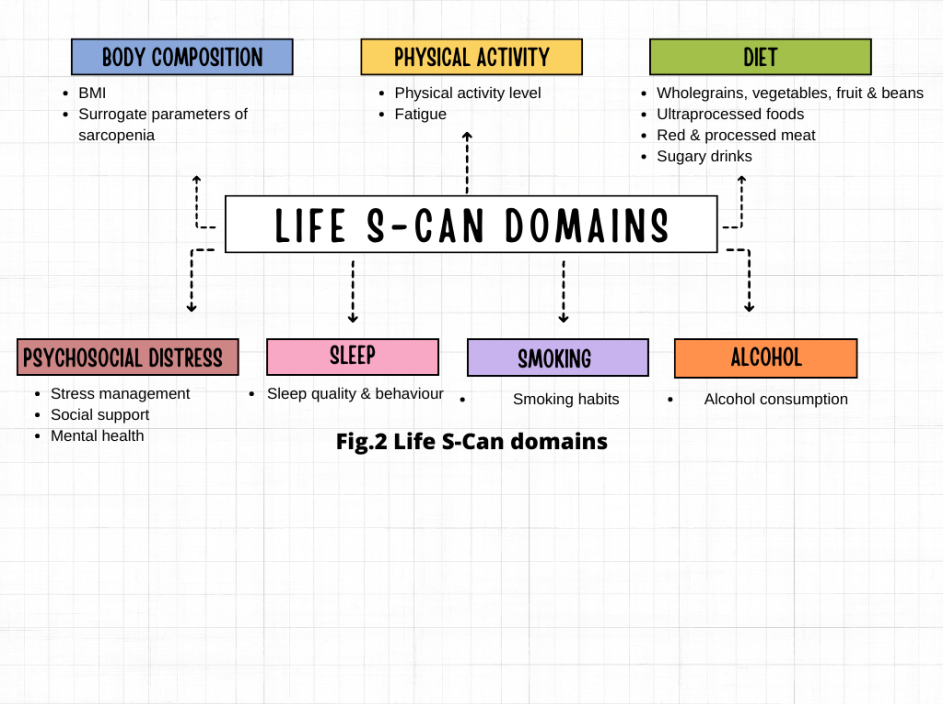
If a participant agrees to participate, they will be given an information sheet (HIP_1), an informed consent (CI_1) and ten saliva collection tubes + instructions to take home. Once the participant has agreed to participate in the study, a research dietitian will contact the person to convene a day and time to meet at the Clinical Trials Unit. During this call, the dietitian will briefly explain what the study will entail and will give detailed instructions on how to collect saliva with the tubes previously administered (Salivette swabs for two days, at five different time points: at waking, 30 min after waking, at noon, at 5pm and anytime between 8-10pm).

**Workflow of the validation study (Study 2):** The validation study will consist into three visits to the research/hospital facility. The **first visit** will be at the Clinical Trials Unit, will take approximately 1 h and will be divided into three parts: 1) Administration of Life S-Can; 2) Administration of validated questionnaires; and 3) Obtain objective measurements of the patient. The **second visit** will also be at the Clinical Trials Unit and will take approximately 10-15 minutes. Participants will be asked to return the accelerometer and complete the Life S-Can questionnaire again (reproducibility). The **third visit** will be at the Nuclear Medicine Imaging Unit at the HUSE, for dual-energy x-ray absorptiometry (DXA) imaging. If possible, the second and third visit will be done on the same day, considering that the research facility is located within the hospital.

**1. Administration of Life S-Can to n=100 cancer survivors:** A research dietitian will collect standard sociodemographic and clinical data of each participant and will then administer the Life S-Can screener. Participants will be offered to complete the Life S-Can questionnaire again after 10 days to test for reproducibility (either by phone or in person, to be convened with the patient), based on the test-retest method.

An individual code will be used for each participant (structure: LS-XXX) when completing all questionnaires, which only the principal investigator and research dietitian will be able to trace to the individual. On all electronic databases used in this project, only the code will be used so no other user can identify the identity of the participant. This same code will be used to mark all biological samples (saliva, blood). All questionnaires will be on an electronic platform and completed electronically.

**2 Administration of validated questionnaires:** To validate the data obtained using the Life S-Can tool, it is essential to collect the same type of data using validated methods. The following validated and extensively used and published questionnaires will be administered for each of the specified domains: body composition (SARC-F, to determine sarcopenia); physical activity (IPAQ); fatigue (QLQ-30); diet and alcohol (a 143-item validated food frequency questionnaire, a semi-quantitative questionnaire which is repeatedly used in epidemiological studies in Spain, such as PREDIMED-Plus); smoking (adapted Spanish questionnaire from the National Statistics Institute); sleep (Pittsburgh Sleep Quality Index); and psychosocial distress (Perceived Stress Scale – stress; QLQ-30 and PHQ-9 – mental health and quality of life –; Multidimensional Scale of Perceived Social Support – social support –). The principle of data minimization established by the Reglamento General de Protección de Datos (Reglamento (UE) 679/2016 del Parlamento Europeo y del Consejo del 27 de abril de 2016), articles 5 & 89, will be taken into account, and thus only the strictly necessary data will be collected.

**3. Obtain objective measurements:**

- **Body weight and height** will be measured by a research dietitian at the Clinical Trials Unit.
- **Physical activity**: Physical activity, sedentary behaviour and sleep quality will be measured using an accelerometer. All participants will be offered to wear an accelerometer for 7 to 10 days. Accelerometry is one of the most reliable techniques for recording and storing the amount and level of physical activity performed by each person and in a given period of time. An accelerometer will be provided to the participant and it will be explained how to use it, and it will have to be returned after 7-10 days. These accelerometers are owned by the NUTRECOR research group and do not imply any added cost for the research group or for the participant. In addition, the use of an accelerometer constitutes a non-invasive technique.
- **Skeletal muscle mass and fat mass** Skeletal muscle mass and fat mass will be determined by a technician from the Nuclear Medicine Imaging Unit using DXA if possible, or using the bioimpedance measurement technique. DXA analysis is of interest to measure skeletal muscle accurately (in this case, for the interest of determining sarcopenia); however, it implicates a small exposure to radiation, similar to that of an X-ray. For this reason, participants will be given the option to either opt out of this procedure and/or analyze body composition via bioimpedance (which is less accurate for muscle mass).
- **Muscle function**: Muscle function will be determined using grip hand strength and the 30-s chair test.
- **Stress** will be measured by cortisol levels in the saliva samples brought in by the patient. Briefly, the salivary samples brought in by the participant will be stored at –80ºC and assayed for cortisol using an enzyme-linked immunosorbent assay kit following the protocol specified by Salimetrics. Samples will be assayed in duplicate.
- **(optional) Blood sample extraction:** All participants will be offered the option to provide a blood sample, which will be centrifuged to obtain blood specimens and will be stored at -80ºC for future studies in this cohort. This will be completely optional and will not determine participant inclusion in the study.

Staff from the Clinical Trials Unit will aid in sample obtention, processing and storing (research technicians) of all biological samples.

**Data integration and statistical analysis:** All data obtained using the questionnaires and the objective measurements will be integrated into a database. Any additional medical data needed will be obtained through medical records (prior consent by the participant). A data management plan following the FAIR principles will be established. Construct validity will be studied using the hypothetical latent structure, structural equation models and psychometric tests. Pearson correlation and Intraclass correlation and Cohen’s Kappa will be used to test the relative validity, reliability and reproducibility of the screener. Internal consistency of the screener will be analysed using Cronbach Alpha.

**PHASE III. PILOT INTERVENTION STUDY**

Phase III is divided into three stages: intervention design, patient recruitment (in combination with Phase II) and group allocation and intervention. Participation in this study will entail visits to the Clinical Trials Unit at IdISBa, located within the Hospital Universitari Son Espases. When possible, visits will be coordinated with other routine appointments at the hospital in order to reduce as much as possible the burden on the participant.

1. **DESIGN OF INTERVENTION**

The intervention will be designed using multidisciplinary approach, whereby knowledge and expertise in oncology, nutrition, epidemiology, psychology and psychometry, validation and implementation will be called upon. In addition, the views and experiences of cancer patients and patient organizations are essential in designing an effective intervention; hence non-scientific authors will be contacted and included in this phase. Close collaboration between patients, organizations, researchers, clinical fellows and institutions is crucial for this project.

The design of the intervention will be developed as follows:

**Recruit an expert panel:** Together with the Principal Investigator, five key personnel have been listed in this project, all of whom carry out research at the Health Research Institute of the Balearic Islands (IdISBa, Spain) together with other duties (university professor, medical doctor). Key personnel not contemplated as researchers in this project include international experts in nutrition and cancer with whom the PI and Dra. Romaguera have previously collaborated with (*detailed below in “Collaborations involved in the project”*), and will be consulted throughout the project. Cancer survivors will also be contacted at this stage (either through patient-orientated organizations or the hospital) to involve them in the design stage.

**Elements of the intervention at discussion:** A series of meetings, semi-structured interviews and panel groups will be planned with expert panel members and non-scientific actors to define the best intervention. Elements to be defined during this stage are: scoring of the individual domains of Life S-Can herein studied and traffic-light system (good, intermediate and non-adherence, which will direct the intervention and health professionals to be visited), monitoring of patients, pipeline, feedback, appointment system, Clinical Trials Unit and data collection.

**Groups of intervention:** A random allocation system will be applied to participants. Participants allocated to the Control Group will receive the first, baseline visit offered to all participants (described in Phase II, validation study) with a trained dietitian, who will administer the Life S-Can questionnaire and a series of validated questionnaires, and then will be given the results of the screener, but no specific feedback will be given other than the standard care and recommendations already provided by the Oncology Department.

The intervention still needs to be defined; however participants allocated to the Intervention Group will receive a baseline visit with a trained dietitian, who will administer the Life S-Can and depending on the score obtained in the domains of diet, physical activity and psychosocial concerns, the participant will likely be offered to see an oncological dietitian (diet domain), a physiotherapist (physical activity) and/or a psychologist (psychosocial distress) for up to three visits (one individual, two group sessions).

All participants will attend a final visit after 3 months to collect data on feasibility, acceptability and efficacy of the intervention.

1. **RECRUITMENT OF PARTICIPANTS**

Cancer survivors (n=100) will be recruited by the Oncology Department at the HUSE as explained in Phase II.

1. **INTERVENTION STUDY**

The intervention study herein proposed will be defined by an expert panel and non-scientific actors as abovementioned; however, we can envision how it may look like. It may consist of 5 visits maximum over 3 months: one baseline visit for all participants, up to three visits for participants from the intervention group (depending on the care plan designed) and one final visit for all participants from both groups. All visits will be carried out at the Clinical Trials Unit at IdISBa (located in HUSE) and will be monthly (one visit per month) (Figure 2). Visits will be coordinated by the PI of the project, together with other team members (Dra. Romaguera, Mar Nafría). Dietary, physical activity and psychologic interventions will be carried out by in-house researchers (trained oncological dietitian – Mar Nafría, and others as part of Dra. Romaguera’s NUTRECOR team) and external health professionals (accounted for in the budget of the obtained funding: INSPIRE (World Cancer Research Fund) and IMPETUS (IdISba, #IMP23/01).


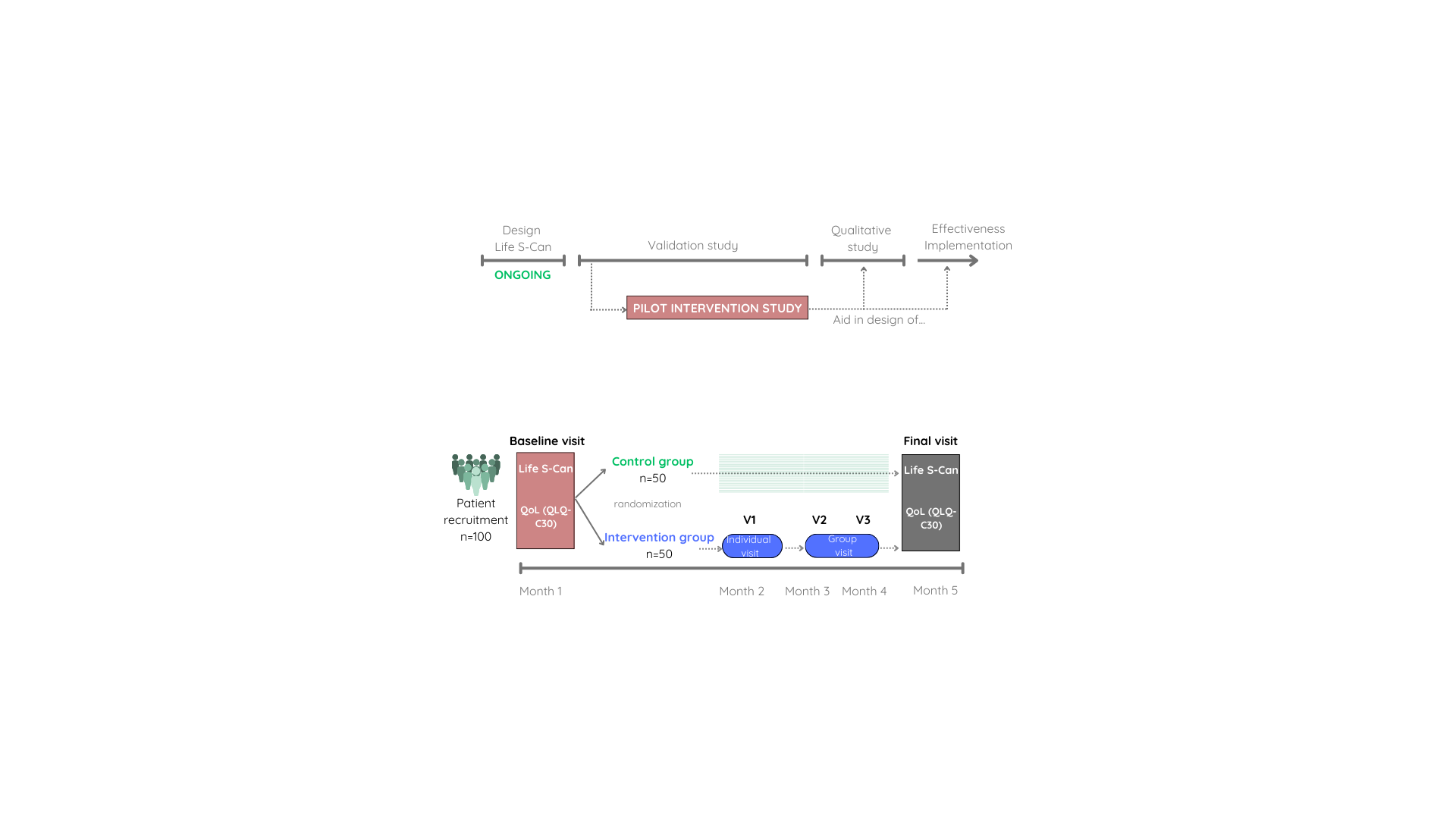


**Figure 2. Intervention study workflow.**

**Baseline visit:** Will be at the Clinical Trials Unit (IdISBa/HUSE), will take approximately 45 min and will be divided into two parts: 1) Administration of Life S-Can; and 2) Administration of the QLQ-C30 for quality of life measurement (together with other validated questionnaires and objective measurements, as part of the Validation Study). All questionnaires will be administered by a health professional using an online platform in Spanish.

Once the baseline visit has been completed, participants will be randomized to either the IG or CG. Once the Life S-Can questionnaire has been administered, it will automatically calculate a score (overall and for each individual domain) and will indicate which domains need improving and to which degree using a traffic light colour system. If the participant is in the control group, they will not receive further advice or care than what they are routinely offered by their oncological care team. Those in the intervention group will be given appointments for visits V1, V2 and V3, according to the score obtained. For example, if a participant scores high for diet but low for physical activity, their care plan will focus on physical activity. Issues will be discussed with the dietitian and a personalised care plan will be devised.

**Visit 1 (V1):** Participants from the IG will be offered a one-to-one session of approximately 30 min with an oncology dietitian, physical activity trainer and/or psychologist, depending on their score (they could be offered only one or all).

**Visit 2 (V2) and visit 3 (V3):** Participants from the IG will be offered group sessions of approximately 1 hour with either an oncology dietitian, physical activity trainer and/or psychologist, depending on their score (they could be offered only one or all).

**Final visit:** Will be offered to all participants (IG and CG), whereby Life S-Can and QLQ-C30 questionnaires will be administered again in order to determine any changes or impact on lifestyle and quality of life. Information regarding feasibility and acceptability will be collected from participants in the IG.

**DATA INTEGRATION AND ANALYSIS**

The last part of this phase will consist in integrating and analysing data obtained from the questionnaires from the baseline visit and the final visit. All data obtained using the questionnaires will be integrated into a database. Any additional medical data needed will be obtained through medical records (prior consent by the participant). A data management plan following the FAIR principles will be established. Expert key personnel listed in this project will be involved at this stage, including Prof. Albert Sesé, a leading expert in psychometric and statistical procedures, and Dr. Ignacio Ricci, with extensive expertise in designing and implementing strategies in health care services. Main outcomes will be measures of feasibility (percentage of patients completing the intervention), acceptability (through ad-hoc questionnaires administered to patients in the IG) and efficacy (improving individual domains of lifestyle and overall QoL). In-house statisticians will be available to aid in data analysis. R, STATA and similar software will be used.

**PHASE IV. QUALITATIVE STUDY:** Phase IV will be divided into three stages (A4.1-A4.3). The aim of the qualitative study (**Study 4**) is to obtain the necessary data to **design a complex intervention to test the feasibility and effectiveness of implementing Life S-Can in a clinical setting**. It is expected to last 2-3 months.

**Design of qualitative study:** Carried out in both a primary care setting and oncology health clinics.

**Participant recruitment and study:** An n=40 participants will be recruited, including cancer survivors, clinicians and health care professionals. Life S-Can will be sent out to all previously by email or phone/message, and four focus groups and semi-structured like in-depth interviews (audio-recorded) will follow. In these, intervention delivery mode, setting, stakeholders involved, expected outcomes and significance of the tool will be assessed. Participants will also be encouraged to share the most important barriers and facilitators for the use of Life S-Can. Both the PI and the research dietitian will be primarily involved in this stage.

**Data analysis:** All interviews will be included in the analysis, will be coded from the audio recordings and analysed using thematic analysis. Two separate researchers will listen to the recordings to annotate the most significant themes discussed.

**8. Legal and ethical aspects**

The study will be carried out in accordance with the principles of the Declaration of Helsinki and the International Conference for Harmonization. All participants will collaborate freely and without any kind of coercion and will be duly informed (Hoja de Información al Paciente studio validación y estudio cualitativo) until they have no doubts about the procedure. They will also receive the informed consent form (Consentimiento Informado estudio validación y estudio cualitativo) that they must sign and obtain a copy of.

The treatment, communication and transfer of personal data of all participating subjects will be in accordance with the provisions of Organic Law 3/2018, of December 5, on the protection of personal data and guarantee of digital rights. According to this law, the participant can access, modify, oppose, cancel, limit the use of and ask for a copy of all their personal data employed in the study. For the validation study, participants’ data and biological samples will be identified by means of a code (LS-XXX) and only the researchers herein mentioned (at the beginning of the document) will have access to them. All data collected by means of forms and questionnaires will be pseudoanonymous (only the PI will have access to the consent forms, saved at IdISBa, planta -1, pasillo G, under lock and key, which will link each participant to their individual code). In the questionnaires, the participant will only have to indicate their individual code and will not be asked for their date of birth, medical history number, name, address, or any other identifying variables. Only sex and age will be recorded. For the qualitative study, participants’ ID will be anonymous since only the type of participant (health professional, patient) will be recorded by the PI and researcher involved (explained in the project), but no other personal data will be collected. Researchers involved will not have access to their medical data and their ID will not transcend to the rest of the research team. All data will be processed and stored on the PI’s computer by the PI and will be incorporated into an automated system that complies with all security measures (coding, passwords) for restricted access to the purpose described in this document.

To guarantee the confidentiality of the information obtained, the data and samples of the patients will be identified by a code and only the study investigator and collaborators will be able to relate said data with the clinical history. Therefore, the identity of the patient will not be revealed to any person except in case of medical emergency, requirement of the Health Administration or legal requirement. Only the essential data necessary to carry out the study will be transmitted to third parties and other countries, and in no case will they contain information that can directly identify the patient, such as name and surname, initials, address, Social Security number, etc. In the event that this assignment occurs, it will be for the same purposes of the study described and guaranteeing confidentiality with at least the level of protection of the legislation in force in our country. The researcher responsible for these data is: Dr. Alice Chaplin Scott, IdISBa (Unidad de Investigación, +34 9871 2050000 ext. 66300). Data will be kept for 10 years.

In the case of biological samples (saliva, blood), these will be obtained and stored according to what is established in Law 14/2007 3^rd^ July of Biomedical research. Biological samples will be stored in a sample collection (ref. C0008029) for 10 years. The study does not include the use of any medication or intervention or change of current treatments. All samples will be identified with a code (LS-XXX). The participant can decide at any time of the study to eliminate or change their consent for the use of their biological samples. Biological samples and the derived data could be passed on to third parties with the aim to participate in national and international research consortia. Data could also be part of the database for the study of genetic factors such as dbGap (<https://www.ncbi.nlm.nih.gov/gap>). In any case, all samples and data will be shared in a confidential manner to fully protect the identity of the participant and will only be used in research projects which have gone through an Ethical Committee review The researcher responsible for these samples is: Dr. Alice Chaplin Scott, IdISBa (Unidad de Investigación, +34 9871 2050000 ext. 66300). Data will be kept for 10 years.

Data derived from this project will be published in international scientific journals to make results available to all the scientific community.
