# Supplementary material for: Validation and pilot feasibility study of a novel screener to assess diet, lifestyle and mental health in people living with and beyond cancer: Study protocols
Source: PLoS One. 2025 Jun 5;20(6):e0323671. doi: 10.1371/journal.pone.0323671 (PMC12140243; doi:10.1371/journal.pone.0323671)
Supplement: S1 Table — (DOCX) [file pone.0323671.s003.docx]

**Supporting Information S3. Ad hoc questionnaire used for the Face Validity Study.**

| The following questions make reference to the questionnaire you have just answered. Your answers will help us refine and improve this questionnaire. Thank you! | |
| --- | --- |
| **Do you believe the questionnaire is written using language which is easy to understand?** | - Yes - No   Please include any other information and/or suggestion that you consider could improve the comprehension of the questionnaire: |
| **Did you need help to answer the questionnaire?** | - Yes - No - If you answered “Yes”, please let us know who helped you (research team, a family member, a friend, a health professional, or others): |
| **Do you believe any of the questions are ambiguous or you were doubtful about?** *For example, it is not clear what is being asked.* | - Yes - No - If you answered “Yes”, please let us know which questions and any additional information you consider could be helpful: |
| **Do you believe the number of available answers is adequate?** *For example, there are not enough options to choose from or there are too many.* | - Yes - No - If you answered “No”, please let us know which questions and any additional information you consider could be helpful: |
| **Do you believe the available answers are ambiguous or you were doubtful about?** *For example, you were not clear what to answer.* | - Yes - No - If you answered “Yes”, please let us know which ones and any additional information you consider could be helpful: |
| **Do you believe that the content of the questionnaire is relevant to a person who has survived a cancer?** | - Yes - Yes, but I believe it is lacking other essential aspects - No - If you answered “Yes, but I believe it is lacking other essential aspects”, please indicate which content you thought was missing: - If you answered “No”, please let us know what content you believe is not relevant |
| **7) Please let us know if you have any additional comments and/or suggestions.** |  |
